# Determination of Phenylurea Herbicides in Water Samples by Magnet-Integrated Fabric Phase Sorptive Extraction Combined with High Performance Liquid Chromatography

**DOI:** 10.3390/molecules30153135

**Published:** 2025-07-26

**Authors:** Natalia Manousi, Apostolia Tsiasioti, Abuzar Kabir, Erwin Rosenberg

**Affiliations:** 1 Institute of Chemical Technologies and Analytics, TU Wien, 1060 Vienna, Austria; natalia.manousi@tuwien.ac.at (N.M.); tsiasioti@chem.auth.gr (A.T.); 2 Laboratory of Analytical Chemistry, Department of Chemistry, Aristotle University of Thessaloniki, 54124 Thessaloniki, Greece; 3 Department of Chemistry and Biochemistry, International Forensic Research Institute, Florida International University, Miami, FL 33131, USA; akabir@fiu.edu

**Keywords:** phenylurea herbicides, magnet-integrated fabric phase extraction, water, environmental analysis, HPLC-DAD

## Abstract

In this study, a magnet-integrated fabric phase sorptive extraction (MI-FPSE) protocol was developed in combination with high pressure liquid chromatography—diode array detection (HPLC-DAD) for the simultaneous determination of five phenylurea pesticides (i.e., chlorbromuron, diuron, linuron, metoxuron, monuron) in environmental water samples. To produce the MI-FPSE device, two individual sol-gel coated carbowax 20 M (CW 20 M) cellulose membranes were fabricated and stitched to each other, while a magnetic rod was inserted between them to give the resulting device the ability to spin and serve as a stand-alone microextraction platform. The adsorption and desorption step of the MI-FPSE protocol was optimized to achieve high extraction efficiency and the MI-FPSE-HPLC-DAD method was validated in terms of linearity, sensitivity, selectivity, accuracy, and precision. The limits of detection (LODs) were found to be 0.3 μg L^−1^. The relative recoveries were 85.2–110.0% for the intra-day and 87.7–103.2% for the inter-day study. The relative standard deviations were better than 13% in all cases. The green character and the practicality of the developed procedure were assessed using ComplexGAPI and Blue Analytical Grade Index metric tools, showing good method performance. Finally, the developed method was successfully used for the analysis of tap, river, and lake water samples.

## 1. Introduction

Phenylurea herbicides (PUHs) are commonly used in agriculture to control weeds both before and after crop emergence in a variety of grain and vegetable crops, such as rice, potato, cotton, corn, and soybean [1,2]. The general formula of these pesticides typically includes a phenyl ring substituted with a urea functional group. These compounds also contain various substituents that determine their chemical properties and biological activity which greatly affect their action [3]. Despite their advantages in enhancing the production, the use of PUHs can have a negative impact since they can persist and accumulate in the environment for a long time [1,2]. At elevated concentrations, PUHs can be toxic to aquatic organisms, birds, and humans, posing significant risks to both ecosystems and public health [1,2,3]. For environmental and food safety, it is necessary to sensitively determine PUHs in environmental samples since pesticides are potential contaminants of surface freshwater [4,5].

For the determination of PUHs in real samples, chromatographic techniques such as high-performance liquid chromatography (HPLC) and gas chromatography (GC) combined with a wide variety of detection systems are typically employed. Among these, HPLC is commonly chosen since GC requires an additional derivatization step as PUHs are thermally labile compounds [2]. As pesticides occur at low concentration levels in real samples, sample preparation is typically required for their simultaneous extraction and preconcentration [6]. However, established sample preparation methodologies are tedious, time-consuming, and, more importantly, expend large quantities of resources that result in the generation of hazardous laboratory waste [7].

Currently, the adoption of greener approaches for pesticide extraction and sample preparation is a major focus of research [8]. In this context, different miniaturized extraction and microextraction techniques have emerged and are used for pesticide extraction. Microextraction is a green alternative to large-scale extraction techniques such as liquid-liquid extraction and solid-phase extraction, due to their miniaturization which results in reduced resources requirement, reusability, and enhanced user safety [9]. Typical examples of such sample preparation techniques include solid-phase microextraction [10], stir bar sorptive extraction [11], magnetic solid-phase extraction [12], dispersive liquid-liquid microextraction [9], and fabric phase sorptive extraction (FPSE) [13]. These techniques comply with many of the principles of Green Sample Preparation (GSP) that were proposed in 2022 by López-Lorente et al. [7]. These principles prioritize the selection of safer chemicals and materials that show renewability, recyclability, and reusability, the minimization of waste generation and energy demand, the miniaturization of sample preparation, the protection of the operator, and the achievement of high sample throughput among others.

Among these techniques, FPSE has attracted the interest of analytical chemists due to its beneficial characteristics. In this technique, a permeable and flexible fabric substrate is coated with a sol–gel organic–inorganic hybrid sorbent. This results in a microextraction device that can be directly immersed into the sample solution and extract the target analytes. A plethora of sorbents with different chemical characteristics (i.e., polar, non-polar, medium polar, ion exchangers etc.) has been developed to extract analytes with different chemical properties [14]. Magnet-integrated fabric phase sorptive extraction (MI-FPSE) is a modification of the conventional FPSE technique that integrates the microextraction and stirring mechanism into one unit. For this purpose, two FPSE membranes are sandwiched together, and a metallic magnetic stirrer is integrated into the device [15]. MI-FPSE techniques offer several notable advantages, including ease of handling, rapid and simultaneous preparation of multiple samples, environmental sustainability, and cost-effectiveness [16]. Both FPSE and MI-FPSE comply with many of the requirements of GSP.

In this work, MI-FPSE was used in combination with high-performance liquid chromatography (HPLC-DAD) for the determination of PUHs in environmental water samples. To the best of our knowledge, no previous protocol employing FPSE or MI-FPSE has been proposed in environmental analysis for the extraction of PUHs, which serves as a significant class of pesticides. For this purpose, different sol-gel coated MI-FPSE membranes were examined, and the sample preparation step was optimized. Accordingly, the MI-FPSE-HPLC-DAD was validated and applied for the analysis of river and lake water samples. The green character and the practicality of the proposed method were also examined using Complementary Green Analytical Procedure Index (ComplexGAPI) [17] and Blue Applicability Grade Index (BAGI) [18].

## 2. Results and Discussion

### 2.1. Optimization of the MI-FPSE Procedure

The main parameters that affect the extraction performance of PUHs from water samples using FPSE were investigated. The variables that were studied included: the sol-gel sorbent and its size, extraction time, sample volume, stirring rate, salt concentration, extraction solvent, volume of the eluent, and elution time. Each factor was independently studied, while the other variables were kept constant. The following conditions were used during method optimization: 1.5 cm × 1.5 cm^2^ FPSE size, 30 min extraction time, 20 mL sample volume, 1000 rpm stirring rate, 0% *w*/*v* NaCl, 1 mL of MeOH extraction solvent, and 5 min elution time.

In order to find the optimum FPSE membrane for the extraction of PUHs from water samples three different sol-gel coated FPSE membranes were examined, with different properties. For this purpose, polar sol-gel carbowax 20 M (CW 20 M), medium polar sol-gel poly (tetrahydrofuran) (PTHF) and non-polar sol-gel octadecyl (C_18_) membranes were evaluated. The experimental results are shown in Figure S1. As can be observed, the highest extraction recovery was obtained using the sol–gel CW 20 M FPSE membrane for all analytes. This can be attributed to the polar characteristics of PUHs due to the presence of the urea functional group. CW 20 M provides different intermolecular interactions including dipole–dipole interaction, hydrogen bonding, and London dispersion towards PUHs, favoring their extraction [19]. The utilization of a polar sol-gel material was expected to result in the highest extraction efficiency due to the possible existence of dipole-dipole interactions and hydrogen bonding forces. Thus, the sol–gel coated CW 20 M cellulose FPSE membrane was finally chosen for further investigation.

The effect of the size of the membranes was also studied, since the sorbent amount can affect the extraction recoveries of the target analytes [16]. For this purpose, four different dimensions were examined: 1.0 × 1.0, 1.5 × 1.5, 2.0 × 2.0, and 2.5 × 2.5 cm × cm, that correspond to 1.0, 2.25, 4.0, and 6.25 cm^2^. As shown in Figure S2, the extraction recoveries were enhanced by increasing the size and thus the amount of sorbent. Further investigation was performed using two circular membranes, stitched to each other, with r = 1 cm that corresponds to 6.28 cm^2^, to fabricate a MI-FPSE device that resulted in higher extraction recoveries. Therefore, the circular membrane of a sol-gel coated CW 20 M cellulose MI-FPSE membrane was finally chosen for the extraction of PUHs from water samples.

#### 2.1.1. Optimization of the Adsorption Step

**Figure 1:**
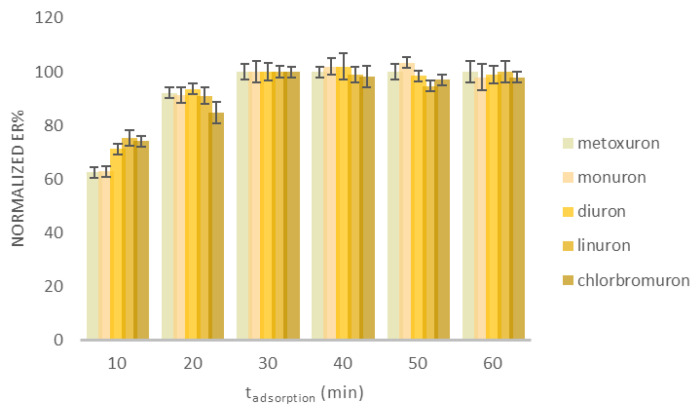
Evaluation of the adsorption time of the FPSE method. Concentration of the selected analytes: 100.0 μg L^−1^ (*n* = 3).

The main variables that affect the adsorption process of the analytes were investigated. The adsorption time was examined at six different values in the range of 10 to 60 min. The results are shown in Figure 1. Since FPSE is an equilibrium-based extraction technique, the optimum adsorption time corresponds to the time that the analytes need to reach the equilibrium between the sample and the sorbent [14]. As can be observed, all analytes reached the equilibrium at 30 min. Any further increase in the adsorption time did not improve the extraction efficiency. Thus, 30 min was selected as a compromise between the sensitivity and the highest sample throughput.

The volume of the sample was investigated, as it affects the extraction recoveries. For this purpose, volumes between 10 and 100 mL were studied, and the value of 10 mL resulted in the highest extraction recoveries, as shown in Figure S3. On the contrary, by increasing the sample volume the preconcentration factors are enhanced (2.5 to 10-fold). For this reason, a volume of 25 mL of sample was chosen as a compromise between the extraction recoveries, the sample consumption, and the preconcentration factors for the selected analytes.

Another important factor investigated was the stirring rate, as it can promote the mass transfer of the analytes to the FPSE membrane, improving the adsorption and thus the extraction recoveries [20]. The stirring rate was examined at four different values in the range 0–1500 rpm. The experimental results are shown in Figure S4. As expected, the extraction recoveries were enhanced after increasing the stirring rate, with the highest values obtained at 1000 rpm. Further increase did not improve the mass transfer of the analytes. Therefore, 1000 rpm was selected as the optimal stirring rate.

**Figure 2:**
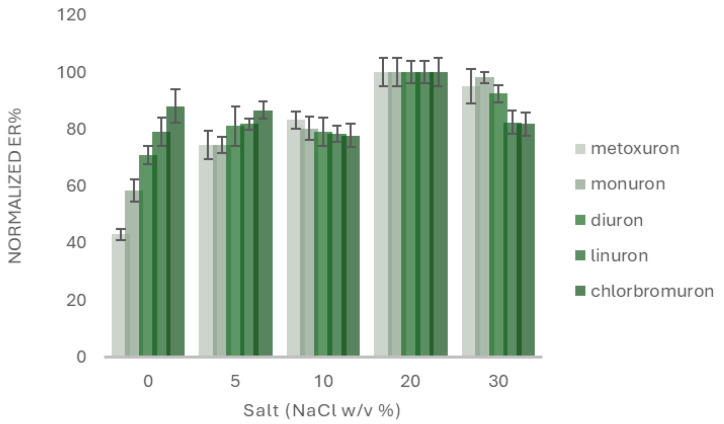
Evaluation of the effect of salt addition. Concentration of the selected analytes: 100.0 μg L^−1^ (*n* = 3).

Finally, the effect of salt addition was studied. For this reason, NaCl was added to the sample, as model electrolyte, in the range from 0% *w*/*v* to 30% *w*/*v*. As shown in Figure 2, the extraction recovery values were increased by increasing the salt concentration. This phenomenon is attributed to the salting out effect, in which the solubility of the analytes is reduced improving the analytes affinity to the sorptive phase [16]. Hence, 20% *w*/*v* NaCl was selected.

#### 2.1.2. Optimization of the Desorption Step

The main parameters that influence the desorption of the analytes from the FPSE membrane were investigated. The elution of the analytes was studied using four different eluents: methanol (MeOH), ethanol (EtOH), acetonitrile (ACN), and acetone (ACE). As shown in Figure S5, MeOH resulted in the most effective desorption of the analytes and was thus selected.

The volume of the eluent is an important factor because it influences the preconcentration factors. The volume of MeOH was investigated at four different values within the range 300–1500 μL. The amount of eluent has to be adequate in order to achieve sufficient desorption and as low as possible to minimize the waste of chemicals [20]. As shown in Figure S6, the extraction recoveries were stable. For this reason, an aliquot of 300 μL of MeOH was chosen, which is in accordance with the principles of green analytical chemistry (GAC).

**Figure 3:**
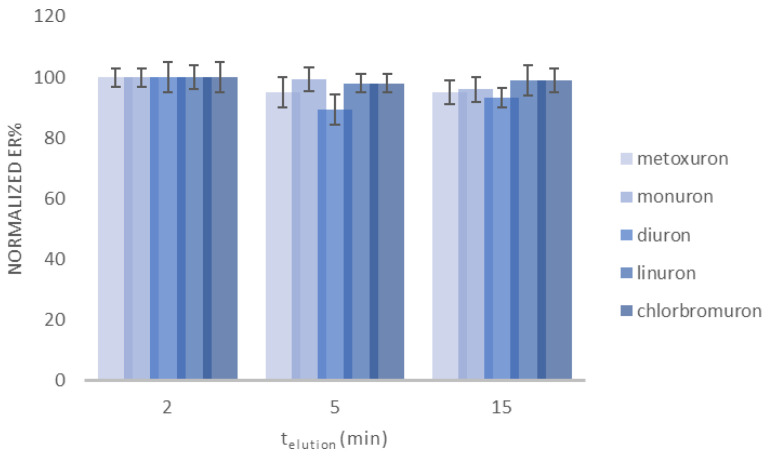
Evaluation of different desorption times. Concentration of the selected analytes: 100.0 μg L^−1^ (*n* = 3).

Finally, optimization of the elution time was conducted in order to find the minimum time span required for sufficient desorption. The elution time was studied between 2 and 15 min and the experimental results are shown in Figure 3. The results were similar at all time spans investigated. Hence the minimum value of 2 min was selected as the optimum elution time to minimize sample preparation time.

### 2.2. Method Validation

The developed MI-FPSE-HPLC-DAD method was validated in terms of linearity, accuracy, precision, limit of detection (LOD), and limit of quantification (LOQ). The analytical figures of merit are shown in Table 1. The linearity was evaluated by using a spiked water sample with the target analytes at concentration levels in the range of 1.0–100.0 μg L^−1^. Least square linear regression analysis was applied to calculate the slopes, intercepts, and coefficients of determination (r^2^) of PUHs. In all cases, the r^2^ value is higher than 0.996, indicating a strong fit to the linear model and thus good linearity for the examined analytes.

The LOQ values were defined as the lowest points of the calibration curves and corresponded to a signal-to-noise ratio of 10. The LOD values corresponded to a signal-to-noise ratio of 3 and were calculated by dividing the LOQ values by 3.3. Thus, the LOQs of PUHs were determined to be 1.0 μg L^−1^ and the LODs were 0.3 μg L^−1^.

The enhancement factors (EF) were calculated by comparing the slope of the calibration curve before and after the preconcentration. The preconcentration factor (PF) was evaluated by dividing the initial volume (25 mL) and the final volume (300 μL) of the sample and was found to be 83. The extraction recoveries were calculated by comparing the EF of the method with the PF of each analyte. The ER% of the PUHs ranged between 51.4 and 61.6%, while their enhancement factors ranged between 42.6 and 51.1.

The precision and the accuracy of the proposed method were evaluated by using spiked water samples at two concentration levels at 5.0 and 50.0 μg L^−1^. The experimental results are shown in Table 2. The intra-day precision was evaluated as the relative standard deviation (RSD%) of five repeated measurements and the inter-day precision by analysis of the spiked samples in three different days. The RSD values ranged from 2% to 7% for the intra-day precision and between 6% and 13% for the inter-day precision. The accuracy of the method was assessed by calculating the relative recovery (RR%), after the comparison of the found and added concentration of the spiked samples. The RR% was satisfactory and ranged from 85.2 to 110.0% for intra-day accuracy and between 87.7 and 103.2% for inter-day accuracy.

The performance of the sol-gel CW 20 M MI-FPSE membrane for the extraction of PUHs was examined as an indicator for the reusability of the membranes. Initially, potential carry-over was checked by using the membrane for the extraction of PUHs at the highest point of the calibration curve, followed by blank analysis. No unwanted carry-over phenomena were observed, indicating that the membrane could be reused. Accordingly, the membrane was repeatedly used for the extraction of PUHs from water at a concentration level of 50.0 μg L^−1^. For the evaluation of the performance, the criterion of ±10% of the RR% was established. The results indicated that the MI-FPSE membrane can be used for at least 15 times, without losing significant performance.

### 2.3. Investigation of Method’s Green Character and Practicality

**Figure 4:**
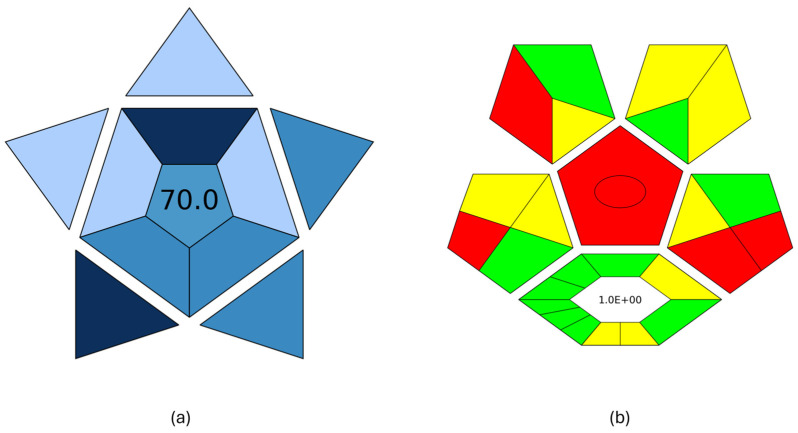
BAGI pictogram (**a**) and ComplexGAPI pictogram (**b**) of the proposed MI-FPSE-HPLC-DAD method.

The practicality of the developed procedure and its green character were examined with BAGI [18] and ComplexGAPI [17] metric tools, respectively. BAGI takes into consideration the main characteristics of an analytical method in terms of sample preparation and analytical determination, and it evaluates its applicability in terms of practicality. This tool generates an asteroid pictogram, and it produces a score that ranges from 25.0 (low practicality) to 100.0 (high practicality). An analytical method is considered practical if it results in a BAGI score higher than 60.0. The MI-FPSE-HPLC-DAD method produced a score of 70.0 (Figure 4a). The good practicality of the developed method can be attributed to the incorporation of different analytes in the analytical method, the simultaneous sample preparation of different samples, the high sample throughput, and the absence of additional steps in the sample preparation procedure. ComplexGAPI evaluates the green character of a method by evaluating the environmental impact of the sample pretreatment steps, the chemicals, instrumentation, sample preparation, and method type, while it can also assess the greenness of the preparation of the new extraction phase used in MI-FPSE. As shown in Figure 4b, the proposed method showed good compliance with greenness requirements due to the selection of a microextraction technique that reduces consumption of chemicals and waste generation.

### 2.4. Comparison with Other Studies

A comparison of the proposed MI-FPSE-HPLC-DAD method with other extraction methods developed for the determination of PUHs in water samples was conducted. As it can be observed in Table 3 the LOD of the proposed method is lower than the respective LOD in refs. [21,22,23], but higher than the methods in refs. [11,24,25,26]. However, it should be noted that the later methods contain an additional evaporation step that enhanced the preconcentration but also the complexity of sample preparation, reducing their practicality. This is clearly reflected in their BAGI scores which ranged between 50.0 and 67.5, which were lower than the value obtained for this study (i.e., 70.0). Due to the evaporation step, ref. [25] shows higher EFs contrary to the developed method, in combination with the high volume of sample applied. Similar EF values are reported for methods in refs. [11,24]. Regarding the accuracy, the present study shows better recoveries in comparison to the most of the works (refs. [11,22,23,24,25,26]). In addition, the herein developed method exhibited comparable precision with the other methods. Finally, the majority of the other methods studied the most common PUHs, mainly monuron and linuron, while chlorbromuron was only included in the present study.

### 2.5. Real Sample Analysis

To demonstrate the practical applicability of the newly developed MI-FPSE-HPLC-DAD method, analyses of various water samples were carried out. Table 4 summarizes the results for real sample analysis. Spiked water samples (50.0 μg L^−1^) were also prepared and analyzed, while the RR% for each analyte was calculated. Since the RR% values were within the range of 86.5–114.3%, the proposed protocol can be used for the reliable determination of PUHs in different surface water samples.

## 3. Materials and Methods

### 3.1. Standards, Chemicals, and Samples

Chlorbromuron, diuron, linuron, metoxuron, monuron (≥ 98.0%) were obtained from Supelco (Bellefonte, PA, USA). Figure S7 shows the chemical structures of the target analytes. For all analytes, a stock solution was prepared in MeOH at a concentration of 1000 mg L^−1^. Working standard solutions were daily prepared in MeOH at appropriate concentration levels.

Methanol (MeOH), acetonitrile (ACN), acetone (ACE), ethanol (EtOH) and water of LC-MS grade were purchased from Honeywell (Charlotte, North Carolina, USA). Analytical grade sodium chloride was purchased from Merck Life Science (Darmstadt, Germany).

Environmental water samples (i.e., lake, river, and tap water) were collected in the municipal area of Vienna, Austria. The collection of the samples was conducted in amber-glass vials with no headspace. Sample containers were stored at 4 °C. No filtration of the samples was necessary prior to their analysis.

For the fabrication of the MI-FPSE device, two sol-gel coated CW 20 M MI-FPSE mebranes were sandwiched together, and a metallic magnetic stirrer was integrated between them. The synthesis of the sol-gel CW 20 M sorbent has been described elsewhere [27].

### 3.2. HPLC-DAD Analysis

**Figure 5:**
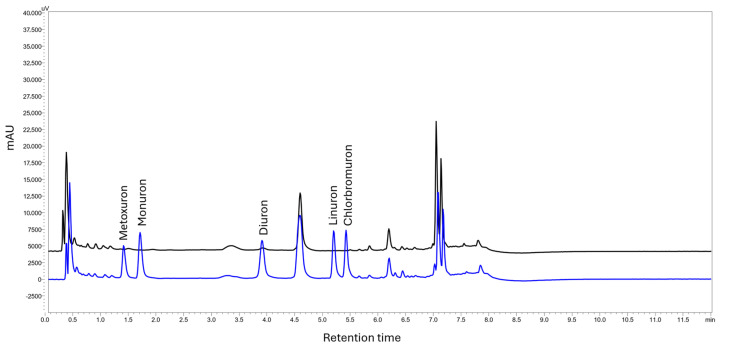
A representative chromatogram of a blank (black) and a spiked (c = 50.0 μg L^−1^) river water sample (blue) analyzed through the proposed MI-FPSE-HPLC-DAD method. Retention times: metoxuron 1.4 min, monuron 1.7 min, diuron 3.9 min, linuron 5.2 min, and chlorbromuron 5.4 min.

An HPLC–DAD system from Shimadzu (Kyoto, Japan) was employed for the quantification of the target analytes. For this purpose, a column oven (CTO-20AC), two pumps (LC-20ADP), a communication bus module (CBM-20A), a degassing unit (DGU-20A3), a diode array detector (SPD-M20A), and an autosampler (SIL-20A) were used. LCMS solution version 3.81 software was used for system handling, data acquisition, and data processing. The separation of the PUHs was performed using a Kinetex C_18_ 100A (2.6 μm, 30 × 4.6 mm) column (Phenomenex, CA, USA). Water (A) and ACN (B) were used as mobile phase. The initial mobile phase composition was 75:25 (A:B, *v*/*v*) and it was maintained for 2.5 min. Then, the composition changed to 55:45 (A:B, *v*/*v*) until 5.0 min and it was kept constant up to 6.0 min. At 6.0 min, the composition was changed until 7.0 min at 80:20 (A:B, *v*/*v*). The system returned to the initial parameters at 8.0 min and it was equilibrated until 12.0 min. The column was thermostated at 30 °C and the analytes were monitored at 245 nm. Using these parameters, the retention times for the analytes were as such: metoxuron 1.4 min, monuron 1.7 min, diuron 3.9 min, linuron 5.2 min, and chlorbromuron 5.4 min. Figure 5 shows a representative chromatogram of the separation of the PUHs. The system suitability parameters are shown in Table S1.

### 3.3. MI-FPSE Procedure

Initially, the MI-FPSE membranes were activated through sequential immersion in 1 mL of an ACN/MeOH (50:50, *v*/*v*) mixture for 5 min and 1 mL of H_2_O for 5 min. For the extraction of the target analytes, the activated membranes were immersed into 25 mL of sample solution (adjusted to 20% *w*/*v* NaCl) that was placed into a 40 mL glass vial. The adsorption took place within 30 min under stirring at a rate of 1000 rpm. Then, the membrane was removed, and the supernatant solution was discarded. The extraction medium was rinsed with water and dried with lint-free tissue paper. The target analytes were eluted by immersing the MI-FPSE membranes in an Eppendorf tube in 300 μL of methanol for 2 min. Following this timespan, the eluate was analyzed by HPLC-DAD. The MI-FPSE membranes were washed with 1 mL of ACN/MeOH (50:50, *v*/*v*), dried, and stored until their next use.

## 4. Conclusions

In this work, a simple and efficient MI-FPSE protocol was proposed for the extraction and preconcentration of five PUHs. The MI-FPSE procedure was combined with HPLC-DAD for water sample analysis. Sol-gel CW 20 M MI-FPSE media showed the highest extraction capability towards the target analytes. No additional pre-treatment (e.g., suspension treatment, centrifugation, filtration) or post-treatment (dilution, evaporation, reconstitution) was required, reducing the steps of analysis, and making the procedure less error prone. The developed method exhibited good performance characteristics in terms of sensitivity, accuracy, and precision. Moreover, the overall scheme exhibited handling simplicity, practicality, green character, and the ability to perform simultaneous sample preparation of different samples. Finally, the MI-FPSE membranes were reusable complying with the requirements of GAC and GSP. The proposed approach could be employed for the extraction of PUHs from other aqueous sample matrices (e.g., fruit juice, tea, herbal infusion samples etc.). However, in this case, the matrix-effect should be assessed, and matrix-matched calibration should be constructed. It is also worth-mentioning that the configuration of MI-FPSE favors the utilization of different FPSE membranes, sandwiched together, for the simultaneous extraction of analytes with a wide range of polarity. All things considered, MI-FPSE can serve as a powerful tool towards greener pesticide determination in environmental and food samples.

## Figures and Tables

**Table 1:** Analytical figures of merit of the proposed MI-FPSE-HPLC-DAD method.

Analyte	Regression Analysis	R^2^	Working Range (μg L^−1^)	LOD (μg L^−1^)	LOQ (μg L^−1^)	ER% ^1^	EF ^2^
Metoxuron	y = 437x + 332	0.9960	1.0–100.0	0.3	1.0	60.3	50.1
Monuron	y = 694x − 92	0.9991	1.0–100.0	0.3	1.0	51.4	42.6
Diuron	y = 765x + 712	0.9968	1.0–100.0	0.3	1.0	61.4	50.9
Linuron	y = 728x + 438	0.9987	1.0–100.0	0.3	1.0	61.6	51.1
Chlorbromuron	y = 701x + 1046	0.9982	1.0–100.0	0.3	1.0	60.5	50.2

^1^ ER%: extraction recovery % ^2^ EF: enhancement factor.

**Table 2:** Accuracy and precision of the proposed MI-FPSE-HPLC-DAD method.

Analyte	Added (μg L^−1^)	Intra-Day (*n* = 5)			Inter-Day (*n* = 3 × 3)		
		Found (μg L^−1^)	RSD%	RR%	Found (μg L^−1^)	RSD%	RR%
Metoxuron	5.0	4.6 ± 0.3	7	91.3	4.7 ± 0.3	7	93.1
	50.0	51.3 ± 1.2	2	105.5	50.5 ± 0.4	8	101.1
Monuron	5.0	4.7 ± 0.3	6	93.3	4.6 ± 0.4	8	92.6
	50.0	55.0 ± 2.0	4	110.0	51.6 ± 0.6	11	103.2
Diuron	5.0	4.8 ± 0.3	6	96.0	4.6 ± 0.3	7	92.8
Linuron	50.0	52.4 ± 1.8	3	104.8	49.1 ± 0.4	9	98.2
	5.0	4.3 ± 0.2	4	85.2	4.5 ± 0.3	7	89.1
	50.0	52.4 ± 0.9	2	104.8	48.4 ± 0.6	12	96.8
Chlorbromuron	5.0	4.3 ± 0.1	3	85.7	4.4 ± 0.3	6	87.7
	50.0	52.1 ± 1.0	2	104.1	47.8 ± 0.6	13	95.6

**Table 3:** Comparison of the proposed MI-FPSE-HPLC-DAD method and with other HPLC methods for the determination of PUHs in water.

Sample Preparation	PUHs	Analytical Technique	Sample Volume (mL)	RR%	RSD%	EF	LOD (μg L^−1^)	BAGI Pictogram	Ref.
Ionic liquid based on dispersive liquid-phase microextraction	Linuron, diuron	HPLC-DAD	10	93.2–98.6	8.68–10.78	N.A. ^1^	0.66–0.74	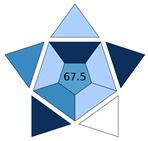	[21]
Supramolecular solvent microextraction	Monuron, linuron, isoproturon	HPLC-UV	20	80.0–99.0	<7.2 (intra) <6.5 (inter)	N.A.	10–30	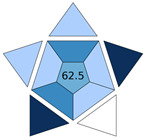	[22]
Automated magnetic micro solid-phase extraction	Monuron, linuron, tebuthiuron, isoproturon,	HPLC-UV	20	80–116	<3 (intra) <6 (inter)	40–57	0.04	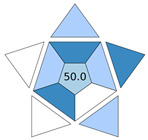	[24]
Functionalized coated stir bar sorptive extraction	Chlortoluron, isoproturon, diuron, linuron	HPLC-DAD	10	80.0–114.8	<4.34 (intra) <8.57 (inter)	46–49	0.025–0.070	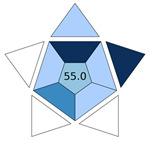	[11]
Magnetic micro solid-phase extraction	Monuron, chlorotoluron, isoproturon, monolinuron, buturon	HPLC-DAD	50	80.6–118.0	<9.6 (intra) <9.6 (inter)	126–227	0.0025–0.015	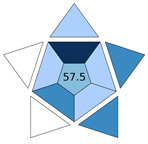	[25]
Magnetic micro solid-phase extraction	Monuron, chlortoluron, monolinuron, isoproturon, linuron	HPLC-UV	10	74.5–111.4	<12.7 (intra) <13.3 (inter)	N.A.	0.06–0.10	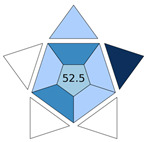	[26]
Solid phase extraction	Monuron, diuron, linuron, metoxuron, metazachlor	HPLC-UV	20	75.0–90.1	N.A.	N.A.	0.82–1.29	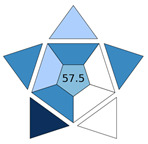	[23]
MI-FPSE	Metoxuron, monuron, diuron, linuron, chlorbromuron	HPLC-DAD	25	85.2–110.0	<7 (intra) <13% (inter)	51.4–61.6	0.3	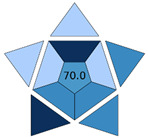	This study

^1^ N.A.: not available.

**Table 4:** Determination of PUHs in water samples through the proposed method (average value ± standard deviation).

Analyte	Added (μg L^−1^)	Tap Water		River Water		Lake Water	
		Found (μg L^−1^)	RR%	Found (μg L^−1^)	RR%	Found (μg L^−1^)	RR%
Metoxuron	-	<LOD	-	<LOD	-	<LOD	-
	50.0	56.0 ± 1.1	112.0	48.4 ± 2.4	96.8	48.0 ± 1.4	96.1
Monuron	-	<LOD	-	<LOD	-	<LOD	-
	50.0	57.1 ± 0.4	114.3	46.3 ± 1.6	92.7	47.8 ± 0.3	95.6
Diuron	-	<LOD	-	<LOD	-	<LOD	-
Linuron	50.0	49.7 ± 3.4	99.5	53.6 ± 3.4	107.2	47.1 ± 3.3	94.1
	-	<LOD	-	<LOD	-	<LOD	-
	50.0	45.3 ± 1.6	90.6	50.1 ± 2.8	100.2	47.5 ± 3.4	94.9
Chlorbromuron	-	<LOD	-	<LOD	-	<LOD	-
	50.0	43.1 ± 2.9	86.5	46.1 ± 1.4	92.2	51.2 ± 3.0	102.4

## Data Availability

Data will be made available upon request.

## Supplementary Materials

The following supporting information can be downloaded at: https://www.mdpi.com/article/10.3390/molecules30153135/s1, Figure S1: Study of different sol-gel membranes. C18: octadecyl, PTHF: poly (tetrahydrofuran), CW 20 M: Carbowax 20, Figure S2: Evaluation of the size of the membrane. Concentration of the selected analytes: 100.0 μg L^−1^, Figure S3: Study of the stirring rate of the proposed MI-FPSE method. Concentration of the selected analytes: 100.0 μg L^−1^, Figure S4: Evaluation of elution solvent of the adsorbed analytes. Concentration of the selected analytes: 100.0 μg L^−1^, Figure S5: Study of different sample volumes. Concentration of the selected analytes: 100.0 μg L^−1^, Figure S6: Chemical structures of the selected analytes, Figure S7: Chemical structures of the selected analytes, Table S1: The system suitability parameters of the proposed MI-FPSE-HPLC-DAD method.

## Abbreviations

The following abbreviations are used in this manuscript:

ACE	Acetone
ACN	Acetonitrile
BAGI	Blue Applicability Grade Index
C_18_	Octadecyl
ComplexGAPI	Complementary Green Analytical Procedure Index
CW 20 M	Carbowax 20 M
EF	Enhancement factor
EtOH	Ethanol
FPSE	Fabric phase sorptive extraction
GAC	Green Analytical Chemistry
GC	Gas chromatography
GSP	Green Sample Preparation
HPLC	High performance liquid chromatography
HPLC-DAD	High performance liquid chromatography-diode array detection
LOD	Limit of Detection
LOQ	Limit of Quantification
MeOH	Methanol
MI-FPSE	Magnet integrated-fabric phase sorptive extraction
PF	Preconcentration factor
PTHF	Poly(tetrahydrofuran)
PUH	Phenylurea herbicide
RR	Relative recovery
RSD	Relative standard deviation
