# Supplementary material for: Determination of Phenylurea Herbicides in Water Samples by Magnet-Integrated Fabric Phase Sorptive Extraction Combined with High Performance Liquid Chromatography
Source: Molecules. 2025 Jul 26;30(15):3135. doi: 10.3390/molecules30153135 (PMC12348110; doi:10.3390/molecules30153135)
Supplement: Supplementary file 1 [file molecules-30-03135-s001.zip › molecules-3772478-supplementary.pdf]

SUPPLEMENTARY INFORMATION

Determination of phenylurea herbicides in water samples by magnet-integrated fabric phase sorptive extraction combined with high performance liquid chromatography

Natalia Manousi [1], Apostolia Tsiasioti [1], Abuzar Kabir[2], and Erwin Rosenberg [1,*]

[1] Institute of Chemical Technologies and Analytics, TU Wien, 1060 Vienna, Austria. Email: natalia.manousi@tuwien.ac.at, tsiasioti@chem.auth.gr, egon.rosenberg@tuwien.ac.at

[2] International Forensic Research Institute, Department of Chemistry and Biochemistry, Florida International University, Miami, FL, USA. Email: akabir@fiu.edu

***** Correspondence: egon.rosenberg@tuwien.ac.at

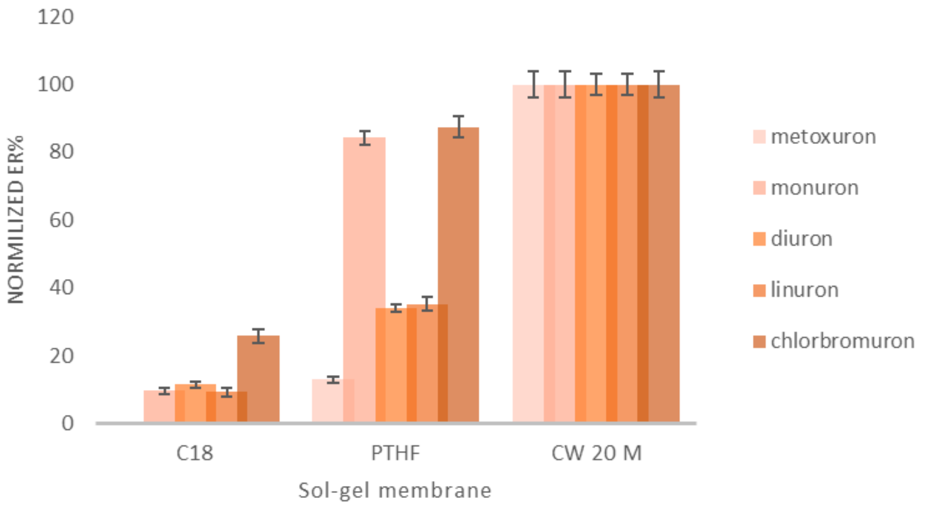

Figure S1. Study of different sol-gel membranes. C18: octadecyl, PTHF: poly(tetrahydrofuran), CW 20 M: Carbowax 20 (n = 3).

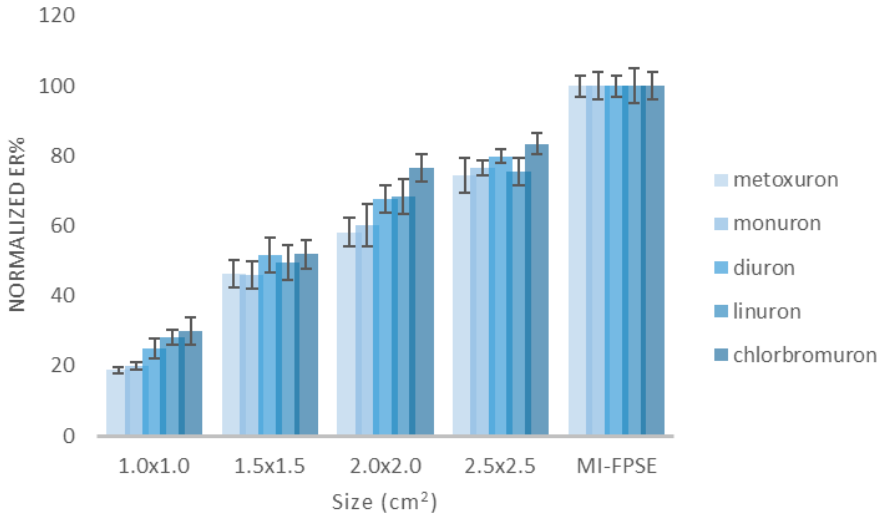

Figure S2. Evaluation of the size of the membrane. Concentration of the selected analytes: 100.0 μg L^{-1} (n = 3).

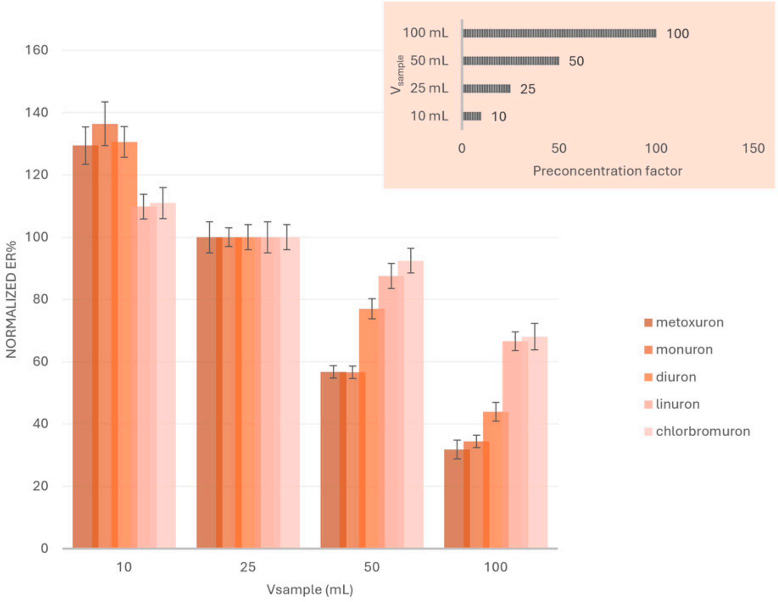

Figure S3. Evaluation of the volume sample and the preconcentration factor. Concentration of the selected analytes: 100.0 µg L^{-1} (n = 3).

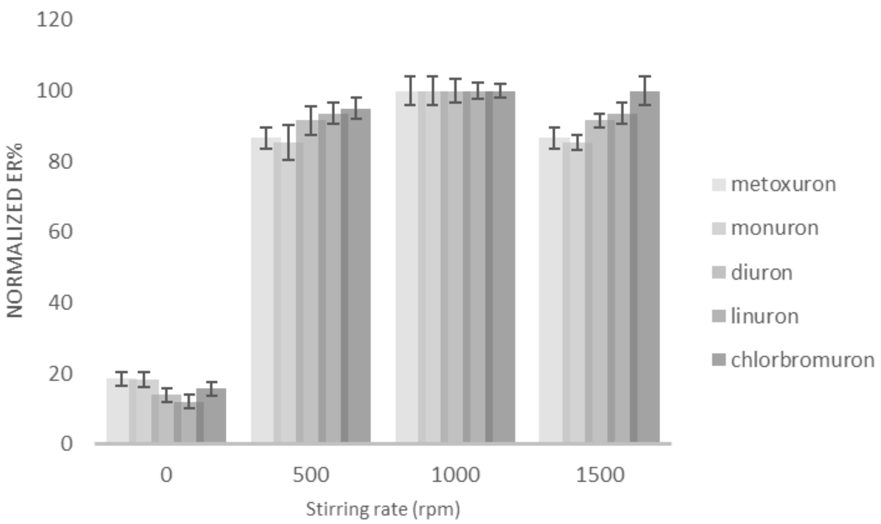

Figure S4. Study of the stirring rate of the proposed MI-FPSE method. Concentration of the selected analytes: 100.0 µg L^{-1} (n = 3).

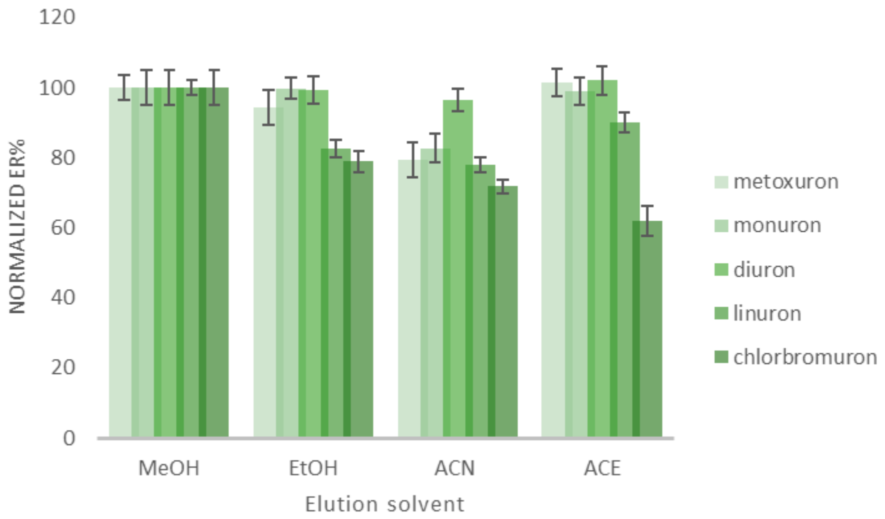

Figure S5. Evaluation of elution solvent of the adsorbed analytes. Concentration of the selected analytes: 100.0 μg L^{-1} (n = 3).

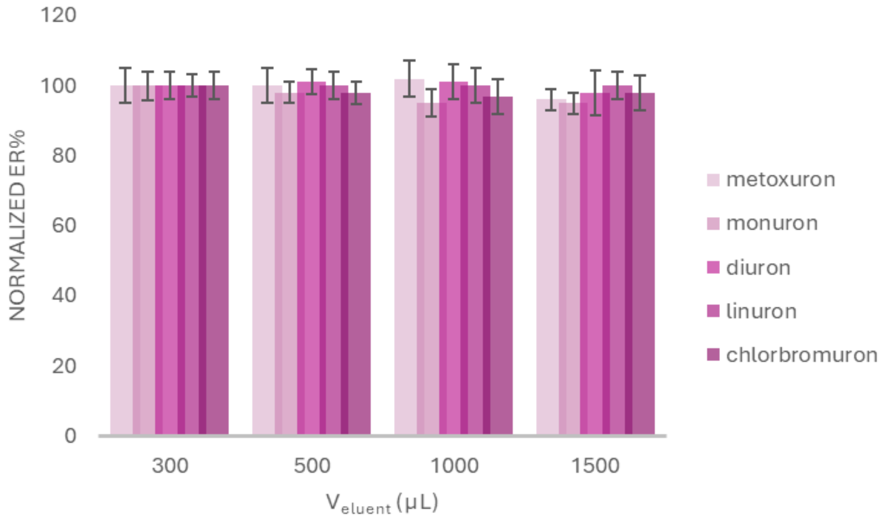

Figure S6. Study of different eluent volumes. Concentration of the selected analytes: 100.0 μg L^{-1} (n = 3).

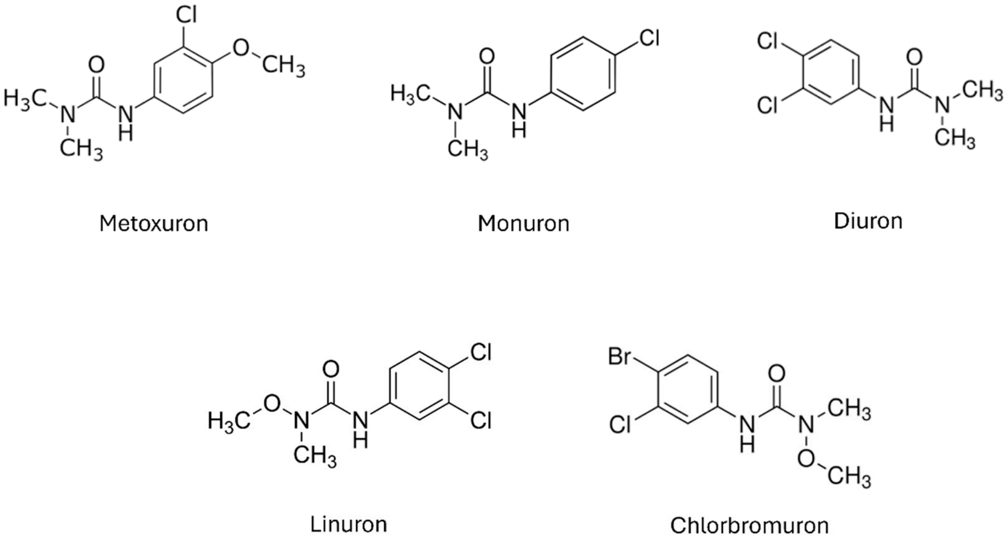

Figure S7. Chemical structures of the selected analytes.

Table S1. The system suitability parameters of the proposed MI-FPSE-HPLC-DAD method.

Analyte	Retention time (min)	Resolution	Tailing Factor	Theoretical Plates
Metoxuron	1.4	-	1.57	1887
Monuron	1.7	2.3	1.66	2537
Diuron	3.9	14.8	1.39	8722
Linuron	5.2	9.2	1.68	31449
Chlorbromuron	5.4	1.9	1.67	36452
